# Aesthetic Preference in the Transverse Orientation of the Occlusal Plane in Rehabilitation: Perspective of Laypeople and Dentists

**DOI:** 10.3390/ijerph182212258

**Published:** 2021-11-22

**Authors:** Ana Lidia Carvalho, Liliana Gavinha Costa, Joana Meneses Martins, Maria Conceição Manso, Sandra Gavinha, Mariano Herrero-Climent, Blanca Ríos-Carrasco, Carlos Falcão, Paulo Ribeiro

**Affiliations:** 1 FP-I3ID, FCS, Universidade Fernando Pessoa, 4249-004 Porto, Portugal; analidiafgdecarvalho@gmail.com (A.L.C.); lilianac@ufp.edu.pt (L.G.C.); joanamenesesmartins@gmail.com (J.M.M.); cmanso@ufp.edu.pt (M.C.M.); sgavinha@ufp.edu.pt (S.G.); cfalcao@ufp.edu.pt (C.F.); ribeiropaulo1@gmail.com (P.R.); 2 FP-I3ID, Instituto de Investigação, Inovação e Desenvolvimento, Universidade Fernando Pessoa, 4249-004 Porto, Portugal; 3 Porto Dental Institute, 4150-518 Porto, Portugal; dr.herrero@herrerocliment.com; 4 Department of Periodontology, Universidad de Sevilla, 41009 Seville, Spain

**Keywords:** prosthodontics, dental esthetics, occlusal plane canting, commissural lines, interpupillary line, facial asymmetry, laypeople, dentists

## Abstract

The present study had a convenience sample with 236 laypeople and 242 dentists who completed an online questionnaire to choose the most attractive image among six pairs for comparison. Control image: symmetric (parallelism between occlusal plane (OP), commissural line (CL), and interpupillary line (IL)). Change of Control, obtaining three images with a 3-degree inclination of the labial commissures. Image A: OP parallel to IL; Image B: OP parallel to CL; Image C: OP at 1.5 degree mean angulation between IL and CL. Non-parametric comparison (IBM© SPSS Statistics vs. 27.0, *p* < 0.05). The “Dentists” group’s decreasing order of preference (attractiveness) of the images is: Control > A > C > B (*p* < 0.05). In the “Lay” group, it is: Control > A > (C not ≠ B). Dentists significantly prefer more the Control and Image A than laypeople (*p* < 0.001). Sex (single exception in laypeople), age, and dentist’s area of activity did not interfere in the perception of attractiveness. Dentists and laypeople preferred the Control when compared to images with CL canted. In the existence of CL inclination, the preference of the groups was the IL as a reference for OP orientation, with the mean angulation or coincident with the CL being considered less aesthetic.

## 1. Introduction

Whenever the rehabilitation of a patient is at stake, an effective integration of the dental prosthesis into the face is a determining factor for achieving a successful final result [1]. To achieve this goal, one of the key factors in determining the prognosis of totally edentulous patients is the establishment of an occlusion compatible with the movements produced by the stomatognathic system [2]. There is also the need to respect the patient’s craniofacial structures and neuromuscular mechanism [3].

One of the three definitions of the occlusal plane (OP) presented in the 9th Edition of the Glossary of Prosthodontic Terms, states that this is “the average plane established by the incisal and occlusal surfaces of the teeth; generally, it is not a plane but represents the planar mean of the curvature of these surfaces” [4]. The correct orientation of the OP results in better stability of the total denture, which allows avoiding the transfer of undue stresses to the underlying residual bone crests, delaying its resorption. Furthermore, it improves the aesthetic performance of the smile and function of the final prosthesis [5,6].

According to Ahmad, facial analysis methods that are based on mathematical principles of beauty assessment, such as anatomical landmarks and imaginary facial lines, are currently used in oral rehabilitation to reestablish OP [7].

The present literature largely explores the principles to be followed by dentists when it comes to reestablishing the OP in a sagittal perspective, valuing the use of different parameters such as the Camper Plan, the Frankfurt Plan, and the Natural Head Position [8,9,10,11].

In order to maintain facial harmony in the transverse analysis of the OP, from a frontal perspective, it is known that it must be perpendicular to the facial midline and parallel to the horizontal facial reference lines, such as the interpupillary line (IL) and the line of the labial commissures (CL) [12,13].

Perfect facial symmetry is a theoretical concept, as all human faces including those considered “most attractive” are asymmetrical [14,15]. Thus, it is difficult for the clinician to establish a threshold that separates pleasant asymmetrical faces from those that need intervention [16,17,18].

Facial asymmetries existing in the CL and the inclination of the OP were described as common conditions that affect the aesthetics of the smile. When faced with these asymmetries, the clinician must choose the reference line with which to guide the transversal OP in the planned oral rehabilitation [19]. However, the relationship between the position of the labial commissures and the OP has not been sufficiently investigated and clarified [1,20].

Currently, the scientific literature disagrees as to which horizontal reference to use in framing the OP in the facial context, and there is also a need to determine within what limits are visually accepted discrepancies in the transverse plane. Thus, the need for further studies to help clarify the dentists’ decision-making process during the rehabilitative diagnosis process is urged [1,12,13,20].

The present study examined what is considered more aesthetic in the opinion of dentists and laypeople when comparing asymmetric smiles to potentially serve as guidance regarding the rehabilitation of patients with CL inclinations and also to investigate the differences in the perception of attractiveness between groups. Finally, it was analyzed if the sex, age, and dentist’s area of activity influenced this perception.

## 2. Materials and Methods

### Data Collection Tool

In this cross-sectional study, data were collected using an online questionnaire consisting of two parts. Initially, socio-demographic issues were addressed (age group, sex, whether layperson or dentist, and in the latter case, the area of activity). Then, the second part included the identification of the preferred image in a total of 6 comparisons of 2 images.

**Figure 1:**
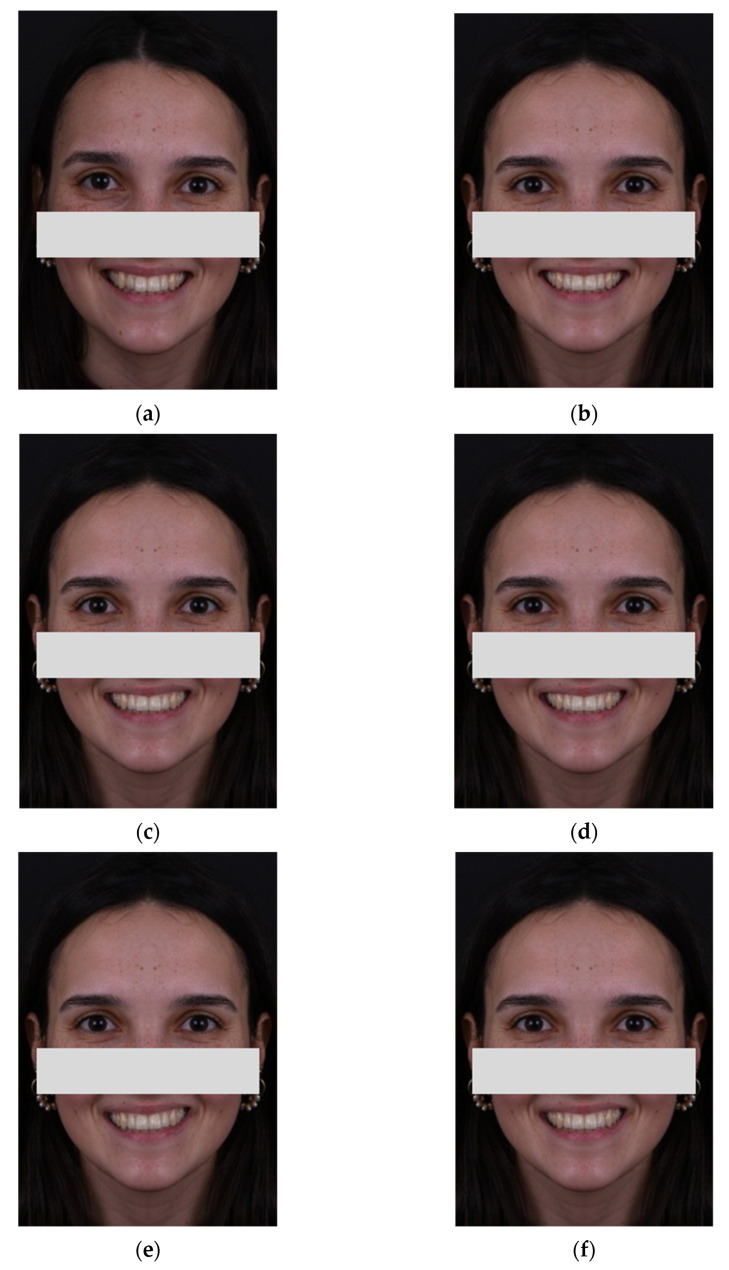
(**a**) Original asymmetric image; (**b**) Symmetrical image—Control; (**c**) 3-degree CL tilt; (**d**) OP parallel to the IL; (**e**) OP parallel to the CL; (**f**) OP mean between the IL and the CL equivalent to 1.5 degrees.

The images used in the questionnaire were obtained from a facial photograph acquired in a photographic studio (Figure 1a), with a Nikon D750© camera (Nikon, Tokyo, Japan) and AF-S VR Micro-Nikkor 105 mm f/2.8 G IF-ED lens (Nikon, Tokyo, Japan). Next, a symmetrical facial model was created (digitally) in which the image designated as Control has parallelism between the OP, the IL, and the CL (Figure 1b). Subsequently, the Control image was altered in order to obtain three new images with a 3-degree CL tilt, counterclockwise (Figure 1c). The 3-degree angle was chosen based on a previous study that found this angle noticeable by most laypersons [19]. These three images differ from each other by the orientation of the OP. Image A has the OP parallel to the IL (Figure 1d), Image B has the OP parallel to the CL (Figure 1e), and Image C has the OP in a mean angulation formed between the angle of the IL and the CL, which is equivalent to 1.5 degrees (Figure 1f). The 4 images (Control, Image A, Image B, and Image C) were arranged in 6 pairs (Control and Image A; Control and Image B; Control and Image C; Image A and Image B; Image A and Image C; Image B and Image C). All manipulations made to the images were carried out in Adobe Photoshop© (Adobe Inc, San Jose, CA, USA) by the main author.

## 3. Process of Editing Images in Adobe Photoshop©

### 3.1. Creation of Symmetrical Facial Model: Control

The facial midline was correctly identified using the program’s ruler. The half of the image that was chosen to be duplicated was selected (rectangular marquee tool). After the selected half was duplicated in a second layer, the images were mirrored to make a single fully symmetrical image. Finally, the two layers were joined to make a single final image. No changes were made to the color and shape of the teeth. The original dental characteristics of the voluntary model were preserved, since maintaining a realistic image was a priority, so as not to cause strangeness or a sensation of artificiality in front of the study participants.

### 3.2. Creation of the Asymmetric Facial Model

The selection of the entire contour of the lip vermilion, inner and outer line was made with the polygonal lasso tool, so that a 3-degree rotation to the top and to the left could be performed (free transformation and rotation). The teeth were not selected and therefore were kept in the same position. Color, texture, fill, and luminosity adjustments were made to correctly adapt the OP, gums, and lips to their new positions (polygonal lasso tool, content aware, mixer brush tool, eyedropper tool, history brush tool). From the asymmetrical facial model, the following 3 images were made with OP deviation.

### 3.3. Creation of Image A: Occlusal Plane Parallel to the Interpupillary Line

Two parallel lines were drawn to be used as a reference in the OP adjustment. One of the lines crossed the two pupils, and the other was rested under the OP. The OP did not need adjustment, as the image was already parallel to the LI.

### 3.4. Creation of Image B: Occlusal Plane Parallel to the Labial Commissures

A line that passed through the two labial commissures was drawn to be used as a reference in the OP adjustment. All teeth were selected at the same time (polygonal lasso tool) from the inner line of the lip vermilion. The selected fragment of the image was rotated 3 degrees to coincide with the left labial commissure (free transformation and rotation). Thus, when tracing the second line under the OP, it was found that both lines were parallel to each other. Color, texture, fill, and luminosity adjustments were made to correctly adapt the OP, gums, and lips to their new positions (polygonal lasso tool, content aware, mixer brush tool, eyedropper tool, history brush tool).

### 3.5. Creation of Image C: Occlusal Plane Mean between the Interpupillary Line and the Commissure Line Equivalent to 1.5 Degrees

All teeth were selected at the same time with the polygonal lasso tool starting from the inner line of the lip vermilion. This selected image fragment was rotated 1.5 degrees toward the left labial commissure (free transformation and rotation). Color, texture, fill, and luminosity adjustments were made to correctly adapt the OP, gums, and lips to their new positions (polygonal lasso tool, content aware, mixer brush tool, eyedropper tool, history brush tool).

## 4. Sample

The participants included in this study were laypeople, as follows: patients from the Pedagogical Dental Clinics of the Universidade Fernando Pessoa and other participants who voluntarily, through a link, agreed to answer the questionnaire shared through social networks. Dentist professors related to the Fernando Pessoa Foundation were also invited to answer the questionnaire as were other dentists who agreed to answer the shared questionnaire through the link in private groups of dentists. All participants had to master the Portuguese language, as the questionnaire was written in Portuguese.

The sizing of the sample (of the two groups) was calculated based on the method of estimation by power analysis for the difference of proportions in two groups, with the following assumptions: the proportion of respondents in a group (Dentist or Lay) that prefers an image was 0.5 (50%); 2% difference in the preference of the two groups was considered as a significant difference between the evaluated groups; a probability of a type I error of 0.05 and a type II error of 20% was considered. In this case, using the expression:

*n* = [*Zα* × √2 × *p* × (1 − *p*) + *Zβ* × √*p*1 × (1 − *p*1) + *p*2 × (1 − *p*2)](*p*1 − *p*2)^2^. (1)

It was calculated that each of these groups should have a size greater than 64 participants (*n* > 64). Data collection took place from February to May 2021, obtaining 478 participants: 236 laypeople (49.4%) and 242 MD (50.6%).

## 5. Ethical Considerations

The study was approved on 10 February 2021 by the Ethics Committee of the Universidade Fernando Pessoa (FCS-MED—127/21) and by the Clinical Direction of the Pedagogical Dental Clinics. Due to the use of a voluntary model for the study, a written authorization was also requested before the images were obtained by camera.

The informed assent filled in by the participant at the beginning of the questionnaire did not imply the collection of his/her name, so it is considered that the questionnaires were filled out anonymously. The complete questionnaire can be read in Supplementary File S1. All the information collected was treated as a group and, in this way, the anonymity of the participants was guaranteed, even in the face of an eventual crossing of socio-demographic data.

## 6. Data Analysis

The data collected from the questionnaires were organized and stored in Excel automatically and the statistical analysis was performed using the IBM© SPSS Statistics vs. 27.0 software (IBM Corp. released 2017, Armonk, NY, USA).

For the purposes of data processing and with regard to the areas of activity of the Dentists, the responses obtained were categorized into two groups: “areas related to aesthetics” and “areas not related to aesthetics”. The first group mentioned above includes Dentistry, Prosthodontics, and Orthodontics, while all other areas were included in the second group (Oral Surgery, Implantology, Oral Medicine, Pediatric Dentistry, Periodontology, Endodontics, Hospital Dentistry, Health Oral Public, and Generalists).

The description of the results obtained was performed using absolute (count of answers) and relative (percentage) frequencies. Chi-square tests were performed to detect significant differences in the choice of the most attractive image by the two groups as well as to identify whether the sex and age of the participants and the area of activity of the dentists were relevant factors in the perception of attractiveness. The comparison of image choice/preference (in each group) was performed using the binomial test. The analysis was performed considering a significance level of 5%.

## 7. Results

It is observed (Table 1) that the decreasing order of preference of the images in the Dentists group is: Control > A > C > B. In the Laypeople group, there is a similar situation; however, no significant difference was detected in the preference (attractiveness) between the B and the C image. Thus, in the Laypeople group, the decreasing order of preference (attractiveness) in the images is Control > A > (C not ≠B). There were also significant differences in the perception of the attractiveness of the images between the groups. In short, Dentists significantly prefer the Control image more than Laypeople did (90.0% vs. 80.9% and 89.3% vs. 73.3%) and the A image more than Laypeople did (86.4% vs. 70.8%).

In Table 2, no significant differences were detected in the preference according to sex (t. Chi-square, *p* > 0.05), except for the comparison between Image A and C for the Lay group (*p* = 0.009), in which women significantly prefer Image A more than men (69.2% vs. 50%).

In the Dentist’s group (Table 2), in both sexes, it is observed that the Control image is significantly more preferred than the A, B, and C images (binomial t, *p* < 0.001 for the three comparisons). In addition, they significantly prefer Image A over Images B and C (*p* < 0.001), but there is no significant difference between Images B and C (*p* = 0.077 and 0.133). Thus, in this group and for each sex, it can be said that the decreasing order of preference (attractiveness) in the images is: Control > A > (C not ≠ B). In the lay group (Table 2), in each sex, the preference is not homogeneous. Thus, for women, the descending order of preference (attractiveness) in the images is: Control > A > (C not ≠B). For lay men, the descending order of preference (attractiveness) in the images is: Control not ≠ A but >C > B, A not ≠C but both >B.

In the results of Table 3, it is observed that in the Dentist’s group, the Control image is significantly more preferred than A, B, and C (*p* ≤ 0.003), and that they prefer A significantly more than B and C (*p* < 0.001), with the exception of the age group “>45 years old” in the comparison between A and C, in which there is no significant difference (*p* = 0.324) in the perception of the images. It is also noted that in the age group of “36 to 45 years”, Dentists significantly prefer C when compared to B (*p* = 0.028).

Thus, in the Dentists group and in the age group “≤ 35 years old”, it can be said that the decreasing order of preference (attractiveness) in the images is: Control > A > (C not ≠B). In the age group from “36 to 45 years old”, the order of preference will be: Control > A > C > B. Finally, the age group “>45 years old” with Control > A > B, but A is not ≠C and B is also not ≠C.

Regarding the Laypeople group, it is observed that only in the age group “≤35 years old” it is possible to establish a descending order of preference (attractiveness) in the images, as follows: Control > A > (C not ≠B). In the age group from “36 to 45 years old”, it was not possible to infer any statistical results due to the insufficient sizing of the sample. In the age group “>45 years old”, only the preference of A was detected when compared to B (*p* < 0.001). Finally, in both groups, no significant differences were detected in preference according to age group.

It is possible to see in Table 4 that the group of areas “not related to aesthetics” detects significant differences in attractiveness in all comparisons made, except between Images B, and C. The Control image is significantly more preferred than Images A, B, and C (*p* < 0.001). Furthermore, there is a significant preference for Image A when compared to Images B and C (*p* < 0.001). However, no significant difference is detected between Images B and C. Thus, for this group, it is possible to state that the decreasing order of preference (attractiveness) in the images is: Control > A > (C not ≠B).

In the group of areas “related to aesthetics”, there are significant differences in attractiveness in all comparisons, with a significant preference for the Control image over Images A, B, and C (*p* = 0.003, *p* < 0.001, *p* < 0.001, respectively). Next, there is a preference for Image A over Images B and C (*p* < 0.001), and significantly more for Image C over B (*p* = 0.012). Thus, in this group, it is verified that the decreasing order of preference (attractiveness) in the images is: Control > A > C > B.

Finally, there are no significant differences between the dentist’s group (areas related to aesthetics and areas not related to aesthetics) in any of the six cases.

## 8. Discussion

The results, when significant, showed a clear preference of dentists and laypeople to the completely symmetrical image when compared to the others, confirming once again the importance of facial symmetry in the perception of attractiveness reported in several studies over the years [21,22,23]. Still, it is important to note that while the brain may receive the image of facial symmetry as a very attractive visualization, all evidence points to varying degrees of asymmetry as the natural state of the human face, both anatomically and functionally [24]. It is up to the dentist to distinguish, from an aesthetic point of view, a pleasing asymmetric face from an asymmetric face that requires intervention [18].

In the present study, the preference found in both groups was for the image with the OP parallel to the IL, even in the presence of deviation in the CL, which goes along with what Ahmad says when he states that: “The interpupillary line is used as a reference for the occlusal and incisal plane orientations. The other horizontal lines can be eschewed and therefore do not act as definite references.” [7]. Even so, this result refutes Silva et al. in their online survey for lay participants when they report that in cases where there is a lack of parallelism between the IL and the CL, most laypeople prefer the transversal OP leaning in the same direction as the commissures [19]. However, the study warns that the degree of OP inclination must be determined for each patient individually, since approximately four out of 10 (40%) participants preferred the OP completely parallel to the IL. The dentists, on the other hand, possibly have their aesthetic perception molded to what is most commonly described in the available scientific literature, in which an example can be given by Fradeani, who defends the concept that the IL, if parallel to the horizontal plane, is the most adequate reference to perform facial analyses. In this context, he adds that ideally, the CL should also be parallel to contribute to general harmony [25]. Finally, a recent study by Farret points out that in cases of asymmetry in the lip architecture when smiling, this asymmetry should not be taken into account when defining the diagnosis and treatment plan; therefore, IL is a more reliable reference in these situations [26].

In cases where there was a difference between the groups in the perception of attractiveness, it was shown that dentists were more capable of opting for the symmetrical image than laypeople, which makes it possible to assume that dentists have more precision in the observation of facial planes and assume them with a higher level of attractiveness, while laypeople tend to be more tolerant. The finding that professionals are able to perceive smaller differences in the OP is consistent with what Revilla-León et al. affirms, which further justifies this result by the fact that dentists are more used to seeing and treating small differences in facial appearance [1]. This finding is repeated in studies that analyzed the OP and other characteristics of the smile [27,28].

Due to the results of a research carried out by Ker et al., it was concluded that although dentists have greater knowledge about the aesthetics of the smile, they should not ignore the fact that laypeople are more tolerant than professionals to the different variations that may exist. The clinician must be careful when identifying the ideal for patients, when this knowledge could sensitize them to unrealistic or unattainable goals [29].

It is also verified in the results that the dentists significantly prefer more than laypeople the OP parallel to the IL when compared to the OP parallel to the CL (with 3-degree inclination), which can be explained by the possibility that the dentists were influenced in their choices in the academic training they had, as explained above, while the laypeople more unpretentiously voted for what seemed most aesthetic to them. Even so, the use of an OP parallel to the IL, as frequently advocated by prosthodontists, can result in suboptimal esthetics in the final restoration if the dentists do not take into account a series of facial asymmetries, in addition to the CL inclination, which can influence the choice of OP during prosthodontic treatment [30]. On the other hand, the use of the labial commissures as a guide for the orientation of the OP has the disadvantage of instability over time, since advancing age causes the corners of the mouth to fall or tilt, and in addition, the commissures are not observed in the same way in dentate and edentulous patients [2]. The dentists still reported more attractiveness in the OP mean between the IL and the CL equivalent to 1.5 degrees than when the OP parallel to the CL (3-degree inclination), while the laypeople were not able to significantly prefer between either of the two options. It is concluded that dentists, when faced with different OP inclinations, preferentially opt for the lowest inclination present in the comparison, not valuing the coincidence between the plane and the CL. As for the laypeople, a possible way of interpreting the observed insufficient preference can be based on the fact that in this group, the participants were possibly not able to distinguish between inclinations present in the two images, allowing the conclusion that inclinations of up to 3 degrees of the OP are not noticed by the laypeople. This contradicts the conclusions of the study by Geron and Atalia, in which it was found for the lay participants that the OP inclination from 2 degrees was perceived and considered unaesthetic [31]. Padwa et al., on the other hand, concluded that laypeople can identify OP angulations greater than 3 degrees 70% of the time [32]. Finally, still equally important to mention, there are two studies that state that laypeople were unable to detect this type of asymmetry up to 4 degrees (in the case of the first study, 3 mm inclinations equivalent to 4 degrees) [27,29]. Attention is also drawn to the fact that these studies did not take into account the relationship between the OP and the CL, which is why it is understood that in these cases, there were only inclinations of the OP, while the CL continued parallel to the IL.

In general, sex was not a factor that affected the perception of attractiveness of each of the groups, which is similarly to the results obtained in the study by Jiménez-Castellanos et al. (for laypeople), in the study by Silva et al. in 2017 (for laypeople), Silva et al. in 2019 (for laypeople), and in disagreement with Revilla-León et al. (for laypeople, dentists, and dental students) [1,12,19,33]. The only exception regarding the participant’s sex occurred in the case of lay women, who significantly preferred the OP parallel to the IL than with a mean between the IL and the CL equivalent to 1.5 degrees, while lay men had no significant results that could declare the existence of some preference. These results can be interpreted in correlation with a study carried out by Koidou et al., in which they state, in agreement with previous findings, that dental appearance is more important for women than for men [23]. Additionally, according to Silva et al., women are recognized as being more critical of beauty and aesthetics than men [22]. Finally, Revilla-León et al. add that men more easily find an image attractive and give higher ratings than women to the same image [1]. Assuming lay women as more demanding than lay men, based on the aforementioned authors, one can try to assume that in the present study, there may have been greater female attention and possibly greater distinction in the details present in the images, resulting in a greater preference for one of the images.

The age of each group was not a factor that affected the perception of attractiveness, since when comparing each pair of images, the age ranges of each group did not differ significantly in their responses. This result is in agreement with what Silva et al. stated when analyzing the perception of laypeople facing OP inclinations along with nose and chin deviations [12]. The opposite was found by Revilla-León et al. since, on a scale from 1 to 6, for the classification of the OP inclination, older people (laypeople, dentist and dental students) tended to give higher ratings, that is, to consider images more attractive than the younger participants did [1].

Even so, in the present study, it is possible to verify that dentists up to 45 years old were more often in agreement with each other, with regard to the perception of attractiveness, than older dentists. In younger laypeople, the situation repeats itself even more clearly. In addition to the difficulty of laypeople distinguishing images with OP inclination of up to 3 degrees, as reported earlier in this Discussion section, a potential explanation for the fact that older people, in general, cannot distinguish images in order to have a preference may have origin, for example, in the senescence process itself. This process is associated with decreased visual acuity, which is caused by the reduction in the ability to focus on objects at close range. According to Wolffsohn and Davies, the prevalence of uncontrolled presbyopia affects up to 50% of people over 50 years of age in developing countries and 34% in developed countries [34]. Furthermore, it is added that older people can be particularly vulnerable to the development of attentional fatigue due to age-related physiological changes [35]. This fact may be related to the reduction in the attention capacity available to deal with a questionnaire, for example. On the other hand, this effect on the results may have been further enhanced by the increased interest in smile aesthetics in new generations of dentists and laypeople, which may lead to more attention and interest in participation in these age groups.

Areas related to aesthetics were not significantly different in the perception of attractiveness when compared to areas not related to aesthetics, which means that the view of professionals in Dentistry, Prosthodontics, and Orthodontics is not substantially different from other dentists. A study from the 1990s reached a similar conclusion, when orthodontists were not shown to be more demanding or observant than other dentists in the face of small discrepancies in the occlusal plane [27]. This result is opposed to that found by Olivares et al. in a study with the objective of determining whether the OP inclination is a factor that influences the aesthetic evaluation of the smile. In this study, a separation was also made between orthodontists and other dentists to assess whether there was a difference in the perception of the attractiveness of the groups, and the results showed that orthodontists considered the OP inclination less acceptable than the other dentists [36]. In addition, another research study found that the increase in the OP inclination and gingival exposure negatively influence the attractiveness of the smile, and even that orthodontists were less generous in the scores they gave to asymmetric smiles than other dentists [37]. Finally, in the study by Dalla-Corte et al., when it came to the OP inclination and mandibular deviation, despite being perceived by both groups, orthodontists showed a greater perception of deviation [18].

## 9. Limitations and Strengths

Ideally, the sample should have been more heterogeneous in terms of age and sex (most participants were young and female). With regard to the areas of activity of the dentists, this study distanced itself from previous studies that prioritized an analysis of the perspective of orthodontists, disregarding other areas of aesthetics, which created a limitation in the comparison of results. These studies also used different types of images (facial/smile photographs) and lack detailed descriptions about their editing, which was necessary to allow a more in-depth comparison of results.

This study aimed to value the opinion of all dentists who work in the field of esthetics and oral rehabilitation. All data collection/analysis and image processing were carefully described to facilitate future investigators who decide to replicate the study with a larger sample.

## 10. Conclusions

Dentists and laypeople preferred the fully symmetrical image (Control) when compared to the other three CL tilted images. In case there is an inclination of the CL, the preference of both groups was to use the IL as a reference parameter in the orientation of the transverse OP, since the mean deviation or deviation coinciding with the CL was considered less aesthetic.

In cases where there were differences in perception between the groups, the dentists significantly preferred more the symmetrical image (when compared to the mean deviation or deviation coincident with the CL) and more the image with the OP parallel to the IL (when compared to the deviation coincident with the CL) than the laypeople.

There were no statistically significant differences in the perception of attractiveness related to sex (except for a single exception in the lay group), age group, or area of activity of the dentists.

## 11. Future Research

Further studies on this topic with a larger and more heterogeneous sample are advised. In addition, it is recommended to evaluate the minor deviations of the occlusal plane in cases of asymmetry of the commissure line for a more in-depth analysis of the visual recognition threshold of dentists and laypeople and the differences in their perception.

## Figures and Tables

**Table 1:** Comparison of the perception of attractiveness in each group and between groups.

Image	Dentists n = 242	Laypeople n = 236	*p* **
Control A *p* *	176 (72.70%) 66 (27.30%) **<0.001**	169 (71.60%) 67 (28.40%) **<0.001**	0.785
Control B *p* *	220 (90.90%) 22 (9.10%) **<0.001**	191 (80.90%) 45 (19.10%) **<0.001**	**0.002**
Control C *p* *	216 (89.30%) 26 (10.70%) **<0.001**	173 (73.30%) 63 (26.70%) **<0.001**	**<0.001**
A B *p* *	209 (86.40%) 33 (13.60%) **<0.001**	167 (70.80%) 69 (29.20%) **<0.001**	**<0.001**
A C *p* *	174 (71.90%) 68 (28.10%) **<0.001**	153 (64.80%) 83 (35.20%) **<0.001**	0.096
B C *p* *	102 (42.10%) 140 (57.90%) **0.017**	107 (45.30%) 129 (54.70%) 0.172	0.482

* Binomial test; ** Chi-square test. Bold *p* values stand for significative differences.

**Table 2:** Comparison of the perception of attractiveness in each group and by sex.

Image	Dentists			Laypeople		
	Female n = 155	Male n = 87	*p* **	Female n = 182	Male n = 54	*p* **
**Control** **A** *p* *	115 (74.2%) 40 (25.8%) **<0.001**	61 (70.1%) 26 (29.9%) **<0.001**	0.494	135 (74.2%) 47 (25.8%) **<0.001**	34 (63.0%) 20 (37.0%) 0.077	0.109
**Control** **B** *p* *	143 (92.3%) 12 (7.7%) **<0.001**	77 (88.5%) 10 (11.5%) **<0.001**	0.330	152 (83.5%) 30 (16.5%) **<0.001**	39 (72.2%) 15 (27.8%) **0.002**	0.064
**Control** **C** *p* *	142 (91.6%) 13 (8.4%) **<0.001**	74 (85.1%) 13 (14.9%) **<0.001**	0.114	138 (75.8%) 44 (24.2%) **<0.001**	35 (64.8%) 19 (35.2%) **0.041**	0.108
**A** **B** *p* *	138 (89.0%) 17 (11.0%) **<0.001**	132 (72.5%) 50 (27.5%) **<0.001**	0.106	71 (81.6%) 16 (18.4%) **<0.001**	35 (64.8%) 19 (35.2%) **0.041**	0.274
**A** **C** *p* *	112 (72.3%) 43 (27.7%) **<0.001**	62 (71.3%) 25 (28.7%) **<0.001**	0.869	126 (69.2%) 56 (30.8%) **<0.001**	27 (50.0%) 27 (50.0%) 1.000	**0.009**
**B** **C** *p* *	66 (42.6%) 89 (57.4%) 0.077	36 (41.4%) 51 (58.6%) 0.133	0.856	88 (48.4%) 94 (51.6%) 0.711	19 (35.2%) 35 (64.8%) **0.041**	0.088

* Binomial test; ** Chi-square test. Bold *p* values stand for significative differences.

**Table 3:** Comparison of the perception of attractiveness in each group by age group.

Image	Dentists				Laypeople			
	≤35 Years Old n = 145	36 to 45 Years Old n = 60	>45 Years Old n = 37	*p* **	≤35 Years Old n = 172	36 to 45 Years Old n = 15	>45 Years Old n = 49	*p* **
**Control** **A** *p* *	105 (72.4%) 40 (27.6%) **<0.001**	43 (71.7%) 17 (28.3%) **0.001**	28 (75.7%) 9 (24.3%) **0.003**	0.903	124 (72.1%) 48 (27.9%) **<0.001**	12 (80.0%) 3 (20.0%) n.a.	33 (67.3%) 16 (32.7%) 0.022	0.614
**Control** **B** *p* *	135 (93.1%) 10 (6.9%) **<0.001**	54 (90.0%) 6 (10.0%) **<0.001**	31 (83.8%) 6 (16.2%) **<0.001**	0.204	142 (82.6%) 30 (17.4%) **<0.001**	13 (86.7%) 2 (13.3%) n.a.	36 (73.5%) 13 (26.5%) 0.002	0.304
**Control** **C** *p* *	133 (91.7%) 12 (8.3%) **<0.001**	51 (85.0%) 9 (15.0%) **<0.001**	32 (86.5%) 5 (13.5%) **<0.001**	0.309	128 (74.4%) 44 (25.6%) **<0.001**	12 (80.0%) 3 (20.0%) n.a.	33 (67.30%) 16 (32.7%) 0.022	0.511
**A** **B** *p* *	126 (86.90%) 19 (13.1%) **<0.001**	52 (86.70%) 8 (13.3%) **<0.001**	31 (83.8%) 6 (16.2%) **<0.001**	0.883	117 (68.0%) 55 (32.0%) **<0.001**	12 (80.0%) 3 (20.0%) n.a.	38 (77.6%) 11 (22.4%) **<0.001**	0.311
**A** **C** *p* *	107 (73.8%) 38 (26.2%) **<0.001**	44 (73.3%) 16 (26.7%) **<0.001**	23 (62.2%) 14 (37.8%) 0.188	0.358	111 (64.5%) 61 (35.5%) **<0.001**	10 (66.7%) 5 (33.3%) n.a.	32 (65.3%) 17 (34.7%) 0.046	0.983
**B** **C** *p* *	66 (45.5%) 79 (54.5%) 0.319	21 (35.0%) 39 (65.0%) **0.028**	15 (40.5%) 22 (59.5%) 0.324	0.373	74 (43.0%) 98 (57.0%) 0.079	8 (53.3%) 7 (46.7%) n.a.	25 (51.0%) 24 (49.0%) 1.000	0.497

* Binomial test; ** Chi-square test; n.a: not applicable. Bold *p* values stand for significative differences.

**Table 4:** Comparison of the dentists’ perception of attractiveness by area of activity.

Image	Area of Activity Related to Aesthetics		
	No n = 158	Yes n = 84	*p* **
**Control** **A** *p* *	120 (75.9%) 38 (24.1%) **<0.001**	56 (66.7%) 28 (33.3%) **0.003**	0.123
**Control** **B** *p* *	145 (91.8%) 13 (8.2%) **<0.001**	75 (89.3%) 9 (10.7%) **<0.001**	0.522
**Control** **C** *p* *	142 (89.9%) 16 (10.1%) **<0.001**	74 (88.1%) 10 (11.9%) **<0.001**	0.671
**A** **B** *p* *	132 (83.5%) 26 (16.5%) **<0.001**	77 (91.7%) 7 (8.3%) **<0.001**	0.080
**A** **C** *p* *	112 (70.9%) 46 (29.1%) **<0.001**	62 (73.8%) 22 (26.2%) **<0.001**	0.630
**B** **C** *p* *	72 (45.6%) 86 (54.4%) 0.301	30 (35.7%) 54 (64.3%) **0.012**	0.139

* Binomial test; ** Chi-square test. Bold *p* values stand for significative differences.

## Supplementary Materials

The following are available online at https://www.mdpi.com/article/10.3390/ijerph182212258/s1, File S1: Parametros Esteticos: Perspetiva De Leigos e de Medicos Dentistas.
