# Supplementary material for: Aesthetic Preference in the Transverse Orientation of the Occlusal Plane in Rehabilitation: Perspective of Laypeople and Dentists
Source: Int J Environ Res Public Health. 2021 Nov 22;18(22):12258. doi: 10.3390/ijerph182212258 (PMC8623891; doi:10.3390/ijerph182212258)
Supplement: Supplementary file 1 [file ijerph-18-12258-s001.zip › ijerph-1437087-supplementary.pdf]

Supplementary Material:

Figure S1. Parametros Esteticos: Perspetiva De Leigos e de Medicos Dentistas.

O presente questionário foi elaborado no âmbito de um projeto de investigação do Mestrado Integrado em Medicina Dentária da Universidade Fernando Pessoa e tem como objetivo avaliar a influência da inclinação da linha média dentária e da inclinação transversal do plano oclusal em faces assimétricas.
Este questionário não deve ser respondido por Estudantes de Medicina Dentária.
É destinado apenas a Médicos Dentistas OU a pessoas que não têm qualquer tipo de conhecimento na área da medicina dentária (leigos), maiores de 18 anos.
O ideal é utilizar um tablet/computador para responder as questões, uma vez que neste questionário é necessário uma observação pormenorizada de algumas imagens. Se estiver a usar o telemóvel, redobre a atenção.
O questionário está dividido em parte 1 e parte 2:

- Parte 1: Será pedido para, escolher entre 2 fotografias a que (na sua opinião) considera com maior nível de atratividade do sorriso.
- Parte 2: Numa primeira fase será pedido para apenas observar atentamente as imagens, e numa segunda fase para classificar o grau de atratividade escala de 0 (nada atraente)-10 (muito atraente) das imagens que vão aparecendo ao longo do questionário.

Não existem respostas certas ou erradas. Responda apenas 1 vez ao questionário.
Este questionário é voluntário, tem o direito de recusar a todo o tempo a sua participação, sem que isso possa ter como efeito qualquer prejuízo pessoal.
O questionário é anônimo, e as respostas às questões serão confidenciais e utilizadas única e exclusivamente para o estudo em causa, sendo guardadas em local seguro durante a pesquisa e destruídas após a sua conclusão/publicação.
Ao prosseguir com o preenchimento deste questionário, declara que assente em participar no estudo em causa, nos termos acima descritos.
Agradecemos, desde já, a sua colaboração.
Caso tenha alguma questão, poderá entrar em contacto connosco através dos seguintes e-mails: 36378@ufp.edu.pt ou 34955@ufp.edu.pt.
Ana Lidia Carvalho
Joana Meneses Martins
*Obrigatório

1. Ao assinalar "SIM" estará a aceitar participar voluntariamente no estudo, permitindo a utilização dos dados fornecidos com o propósito de aumentar o conhecimento científico, confiando que estes apenas serão utilizados para esta investigação e nas garantias de confidencialidade e anonimato. *

   Marcar apenas uma oval.

   - SIM, aceito participar no estudo de acordo com a informação acima facultada.
   - NÃO pretendo participar no estudo.

Questionário:

2. Qual é a sua faixa etária? *

   Marcar apenas uma oval.

   - De 18 a 25 anos
   - De 26 a 35 anos
   - De 36 a 45 anos
   - De 46 a 55 anos
   - De 56 a 65 anos
   - A partir dos 65 anos

3. Qual é o seu Género? *

   Marcar apenas uma oval.

   - Feminino
   - Masculino

4. É: *

Marcar apenas uma oval.

- ⬭ Médico Dentista
- ⬭ Leigo *Pular para a pergunta 6*

É Médico Dentista

5. Qual é a principal área da Medicina Dentária a que se dedica? *

Marcar apenas uma oval.

- ⬭ Cirurgia Oral
- ⬭ Odontopediatria
- ⬭ Ortodontia
- ⬭ Periodontologia
- ⬭ Endodontia
- ⬭ Prostodontia
- ⬭ Medicina Dentária Hospitalar
- ⬭ Saúde Pública Oral
- ⬭ Outro: ______________________

6. Na sua opinião, qual sorriso considera mais atrativo? *

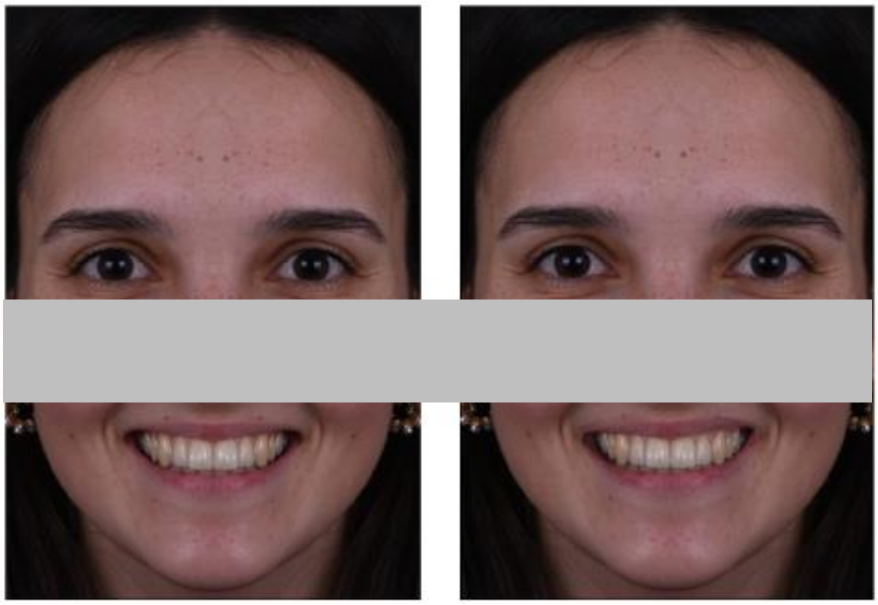

Opção 1 **Opção 2**

Marcar apenas uma oval.

- ⬭ Opção 1
- ⬭ Opção 2

7. Na sua opinião, qual sorriso considera mais atrativo? *

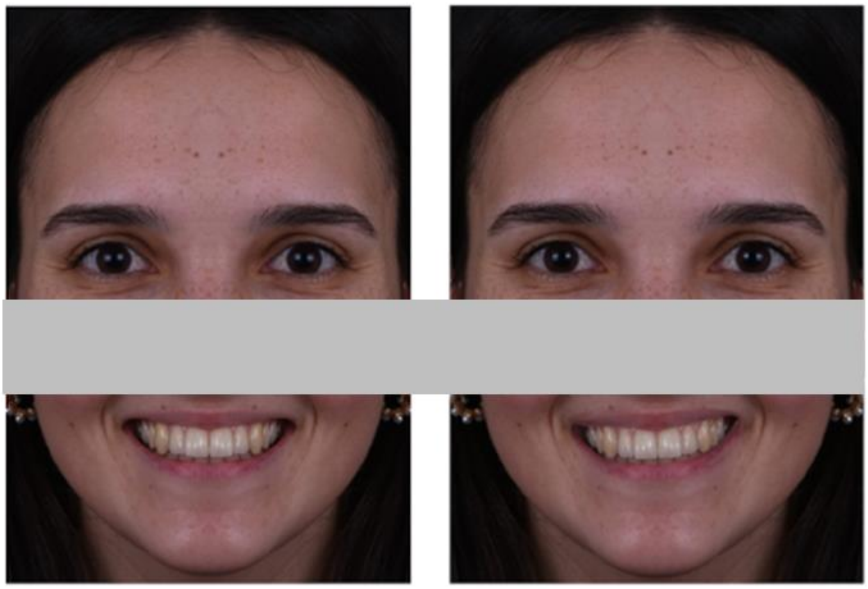

Opção 1 **Opção 2**

Marcar apenas uma oval.

- ( ) Opção 1
- ( ) Opção 2

8. Na sua opinião, qual sorriso considera mais atrativo? *

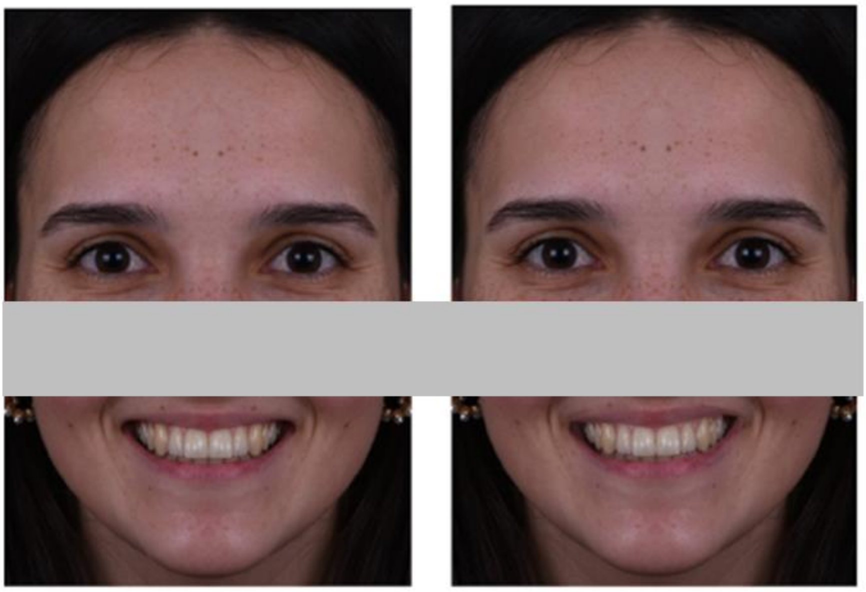

Opção 1 **Opção 2**

Marcar apenas uma oval.

- ( ) Opção 1
- ( ) Opção 2

9. Na sua opinião, qual sorriso considera mais atrativo? *

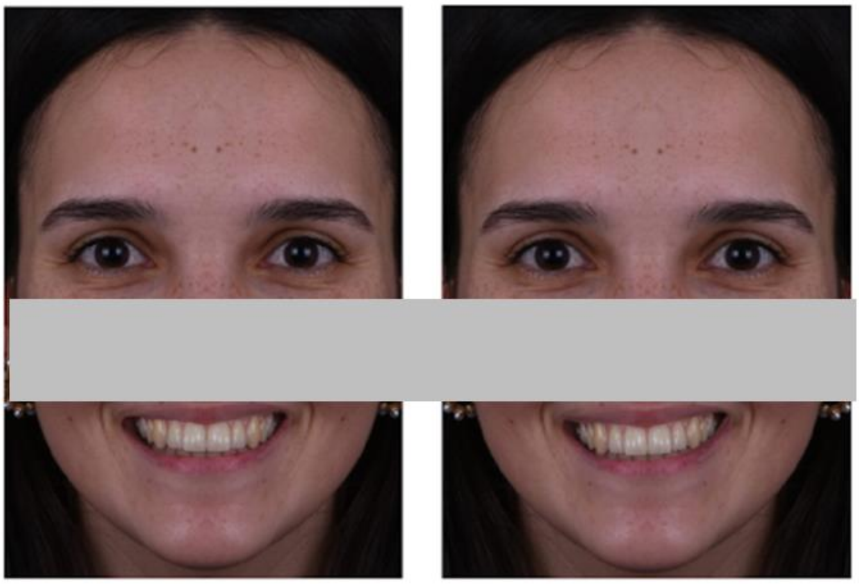

Opção 1 **Opção 2**

Marcar apenas uma oval.

- Opção 1
- Opção 2

10. Na sua opinião, qual sorriso considera mais atrativo? *

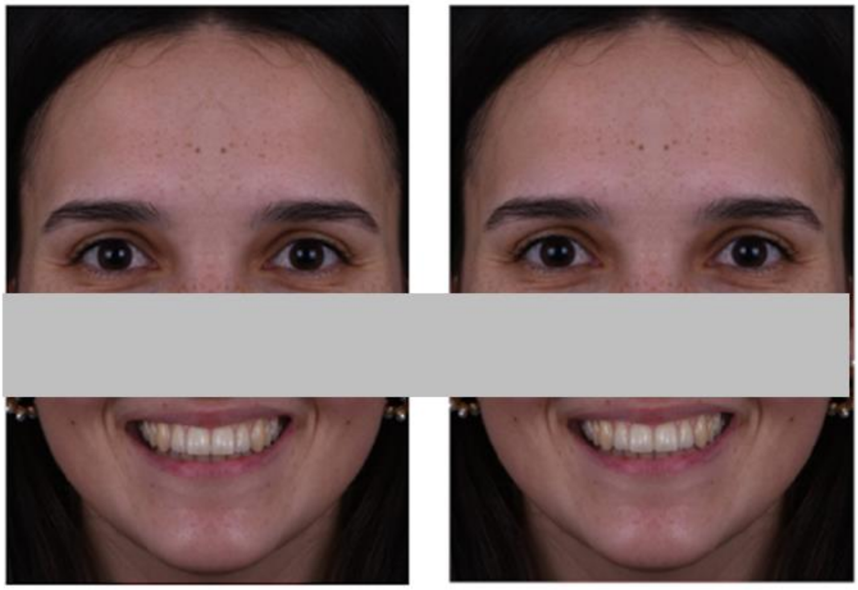

Opção 1 **Opção 2**

Marcar apenas uma oval.

- Opção 1
- Opção 2

11. Na sua opinião, qual sorriso considera mais atrativo? *

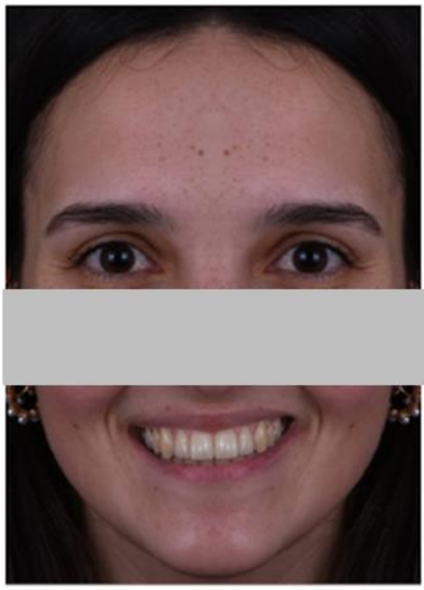

Opção 1

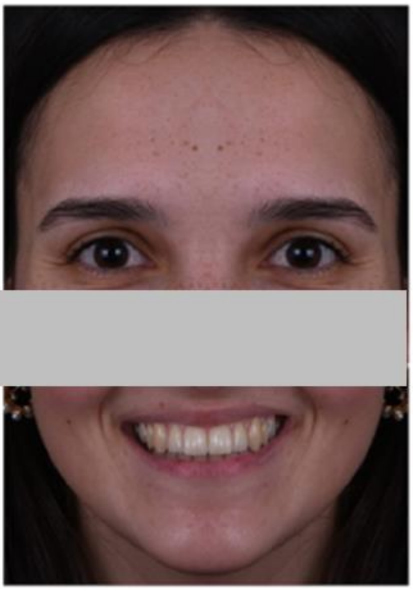

Opção 2

Marcar apenas uma oval.

- ⬭ Opção 1
- ⬭ Opção 2
